# Socioeconomic Differences in Parenting Strategies to Prevent Adolescent Smoking: A Case Study from the Netherlands

**DOI:** 10.1007/s12529-016-9543-1

**Published:** 2016-02-16

**Authors:** Mirte A. G. Kuipers, Sylke Haal, Anton E. Kunst

**Affiliations:** Department of Public Health, Academic Medical Center, University of Amsterdam, P.O. Box 22660, 1100 DD Amsterdam, The Netherlands

**Keywords:** Anti-smoking parenting strategies, Parenting, Smoking, Adolescents, Socioeconomic inequalities

## Abstract

**Purpose:**

This study aimed to identify possible socioeconomic differences in the use of anti-smoking parenting strategies.

**Methods:**

In 2012, survey data of adolescents (*N* = 225) aged 13 to 17 years and their mothers (*N* = 122) and fathers (*N* = 105) were collected in Haarlem, the Netherlands. Questions on smoking behaviour and eleven anti-smoking parenting strategies were answered by adolescents, mothers and fathers. School tracks of adolescents and educational level of parents were measured as indicators of socioeconomic position. Linear multilevel regression analyses were applied to study the association between socioeconomic position (SEP) and standardised scores of anti-smoking strategies. Analyses were controlled for age, sex and smoking by parents and adolescents.

**Results:**

We found no consistent socioeconomic differences in the use of anti-smoking parenting strategies. There were no statistically significant differences in relation to parental educational level or when using adolescent reports on parenting practices. However, when using parental reports, a few strategies varied significantly according to adolescent educational track. Adolescents in higher educational tracks were more likely to have no-smoking rules in the home (standardised regression coefficient (*β*) = 0.20, 95 % confidence interval (CI): 0.03; 0.37, *p* = 0.022) and more likely to have a no-smoking agreement (*β* = 0.17, 95 % CI: 0.00; 0.34, *p* = 0.048). However, they were less likely to frequently communicate about smoking with their parents (*β* = −0.25, 95 % CI: −0.41; −0.08, *p* = 0.004).

**Conclusion:**

In this specific population, there was no consistent support for the hypothesis that anti-smoking parenting strategies contribute to socioeconomic inequalities in adolescent smoking. Parental factors that are more likely to contribute to these inequalities include parental smoking and parenting styles.

## Introduction

Smoking typically finds its origins in adolescence, with approximately two thirds of smokers initiating smoking before the age of 18 [1]. Those who initiate smoking at an early age are more likely to continue to smoke during their adult life [1], leading to health problems such as impaired fitness, increased rates of illness and reduced lung growth at young age, and development of illness such as cancer, COPD and cardiovascular disease in later life [2]. Eventually, smoking kills up to half of its users [3]. The prevention of smoking in young people is crucial in order to reduce this high burden of tobacco-related death and illness.

Adolescents of lower socioeconomic position (SEP) are typically more likely to smoke than their peers from more advantaged backgrounds [4, 5]. They are more likely to initiate smoking and to become regular smokers after having started smoking. These inequalities in smoking may increase in adult life due to individuals of low socioeconomic status being less likely to quit smoking [6]. To prevent large smoking inequalities in adult life, effective strategies are needed to keep adolescents of low SEP from initiating smoking.

Socioeconomic inequalities in adolescent smoking are in part attributable to factors related to their parents. Since adults of low socioeconomic status are more likely to smoke than those of high socioeconomic status [7], children of low SEP more often have parents who are smokers. As parents are strong role models in the lives of young people, adolescents with smoking parents are more likely to take up smoking themselves [8]. Moreover, adolescents of low SEP are more likely to be friends with peers whose parents smoke, and they may therefore be indirectly influenced by the smoking behaviour of peers’ parents [9].

Parents might also influence the smoking behaviour of their children through their parenting practices. A majority of adolescents expects their parents to take action in order to prevent them from smoking [10]. Several anti-smoking parenting strategies may prevent adolescents from initiating smoking [11–17]. These strategies may be intended, such as promising a reward if children do not smoke up to a certain age, but also unintended, such as the reaction parents have when finding out that their child has tried smoking.

The effectiveness of anti-smoking parenting strategies is assessed in many studies (e.g. by Harakeh et al. [11], Abar et al. [12] and Calafat et al. [13]). However, to our knowledge, only one study investigated socioeconomic differences in the use of anti-smoking parenting strategies [14]. Ringlever et al. [14] found that fathers in lower occupational classes communicate with the lowest quality about smoking, while mothers in higher occupational classes communicate most frequently. However, the study did not assess socioeconomic differences in other anti-smoking parenting strategies, and it only focused on socioeconomic characteristics of the parents but not of the children.

The aim of the current study was to identify educational differences in the use of anti-smoking parenting strategies in a sample of secondary school students in Haarlem, the Netherlands. We measured the use of such strategies as reported by adolescents and their parents. We assessed differences according to both the education of the parents and the adolescent. Moreover, we studied a comprehensive set of eleven different anti-smoking parenting strategies.

We hypothesised that higher educated parents and parents of adolescents in higher educational tracks more often used various anti-smoking parenting strategies. We expected to find this pattern according to both adolescents’ and parents’ reports. If so, this would support the hypothesis that anti-smoking parenting strategies contribute to socioeconomic inequalities in adolescent smoking.

## Methods

### Design and Study Population

Survey data were collected in a secondary school in the city of Haarlem, the Netherlands. Haarlem is a medium-sized city (153,100 inhabitants in 2012), with a mean annual income close to the Dutch average (16,200 euros in Haarlem vs. 15,100 euros in the Netherlands, in 2012) [18]. Haarlem is generally considered to be representative for the Netherlands in terms of culture, social structure and history [19].

The study population consisted of 452 individuals: 225 students aged 13 to 17 years, 122 of their mothers and 105 of their fathers. Students were enrolled in the third grade of secondary school. All 225 adolescents who were invited to participate completed the survey; there were no refusals. Adolescents received a blank envelope including one adolescent questionnaire and two parent questionnaires, all with the same serial number. The adolescent questionnaire was completed in classrooms, under the supervision of a teacher and a researcher. The parent questionnaires were taken home by the adolescents for the parents (including, if applicable, step-parents or other adult guardians) to complete. Parents were informed beforehand via e-mail. Completed parent questionnaires were collected in a closed box at the school administration. Ninety-eight adolescents did not provide parent questionnaires, 27 students provided one parent questionnaire (22 with mother only and 5 with father only), and 100 students provided questionnaires completed by both parents. All 225 adolescents were included in the analysis. For the 98 adolescents whose parents who did not complete a questionnaire, their parents were excluded from all analyses.

The Medical Ethics Review Committee of the Academic Medical Center in Amsterdam confirmed that the Dutch Medical Research Involving Human Subjects Act does not apply to the current study and that an official approval of this study was therefore not required (reference number W12_274 # 12.17.0313).

### Measures

#### Anti-Smoking Parenting Strategies

The use of anti-smoking parenting strategies was the outcome variable of interest. We measured eleven strategies with one statement each in all 452 adolescents and parents. Statements as presented in Table 1 were included in the adolescent questionnaire and were rephrased to fit the context of parents in the parent questionnaire. In Table 1, we refer to previous studies using the same or similar statements to measure the same concepts. All included strategies refer to actions that parents may undertake such actions to influence the smoking behaviour of their child or to be more closely involved in their child’s smoking behaviour. Answers to the statements were given on a 5-point Likert scale, with 1 representing ‘strongly disagree’ and 5 representing ‘strongly agree’. We created a total anti-smoking strategy score by computing the mean of all items. Cronbach’s alpha was 0.74. For each parenting strategy, information was missing for at most 1 % of all individuals (i.e. 5 or fewer out of 452 individuals).

**Table 1:** Statements used to measure anti-smoking parenting strategies and means of adolescent and parent reports

Strategies	Statements	Mean (95 % CI)^a^	
		Adolescents (*N* = 225)	Parents (*N* = 227)
Communication quality	Me and my parents are interested in each other’s opinions on smoking [11, 15, 16].	2.37 (2.23; 2.52)	3.40 (3.27; 3.52)
Monitoring child	My parents would know if I would smoke or would experiment with smoking [16, 20, 21].	3.47 (3.31; 3.63)	3.70 (3.59; 3.81)
Monitoring friends	My parents would know if my friends were smokers [20, 21].	3.36 (3.20; 3.51)	3.35 (3.23; 3.47)
Perceived influence	My parents can prevent me from smoking [11, 16, 20, 21].	3.10 (2.94; 3.26)	2.80 (2.66; 2.93)
Negative reaction	If my parents would find out that I smoke, they would be upset and would correct me [16, 20, 21].	3.58 (3.43; 3.74)	3.01 (2.87; 3.15)
Home rules	We have clear smoking rules in our home [16, 21].	3.60 (3.42; 3.77)	4.15 (4.03; 4.28)
Warnings	My parents warn me for the dangers and disadvantages of smoking [20, 21].	3.76 (3.61; 3.91)	4.27 (4.18; 4.36)
No-smoking agreement	I have an agreement with my parents that I will not initiate smoking [11].	3.54 (3.36; 3.72)	3.17 (3.02; 3.32)
Reward	I will be rewarded if I will not start smoking [16].	2.38 (2.18; 2.57)	2.31 (2.16; 2.47)
Communication frequency	I often talk with my parents about smoking related issues [17].	2.44 (2.30; 2.59)	3.12 (2.99; 3.24)
Reacting to smoking	If my parents would find out that I smoke, they would do something about it [20, 21].	3.90 (3.75; 4.04)	4.09 (3.97; 4.20)
Total anti-smoking strategy score	Mean score over all statements	3.23 (3.14; 3.32)	3.27 (3.22; 3.33)

All items were scored on a scale of 1–5, with 1 representing ‘strongly disagree’ and 5 representing ‘strongly agree’

^a^Mean scores with 95 % confidence intervals

#### Socioeconomic Position

We measured the SEP according to the educational track of the adolescents and the educational level of both parents. The school included the three main educational tracks that are distinguished throughout the Dutch educational system: ‘low’ (lower-level secondary education), ‘middle’ (higher-level secondary education) and ‘high’ (pre-university secondary education). Allocation to educational tracks typically occurs at age 12 and is based on the academic performance of students in primary school and early secondary school. The educational track of each student was provided by the school for all adolescents. In the data, the educational track was assigned to the adolescents and to their parents.

Parental educational level was measured in the parent questionnaire and was divided into ‘low’, ‘middle’ and ‘high’. Low educational level included primary school and lower-level secondary education. Middle-level education included vocational education and higher-level and pre-university secondary education. High-level education included higher professional education and university. The higher level of either parent was assigned to both parents and their child. No mothers and only one father did not report their educational level, resulting in 252 valid reports of parental educational level.

#### Smoking Status

Smoking status of adolescents was measured by asking ‘Do you smoke?’, with five response categories: ‘daily’, ‘weekly’, ‘monthly’, ‘tried a few times’ and ‘never’. In the sensitivity analysis, never-smokers were compared with ever-smokers. Smoking status of the parents was measured in the adolescent questionnaire. Adolescents were asked: ‘Do your parents smoke?’, with answer categories: ‘neither’, ‘one of them’ and ‘both’. There were no missing values in the adolescent reports on adolescent and parental smoking. Adolescent and parental smoking statuses were assigned to adolescents and both their parents.

#### Demographics

Age (in years) and sex (boys vs. girls) of the adolescent were measured in the student questionnaire and were assigned to adolescents and both their parents.

### Statistical Analysis

The association between SEP and anti-smoking parenting strategies was investigated using multilevel linear regression analyses with a random intercept at the family level. Families included one adolescent, and a zero, one or two parents. Anti-smoking parenting strategies were the dependent variables, with a higher score representing higher agreement regarding the use of the respective strategy. In the main analyses, we studied differences in parenting strategies according to the educational track of the adolescent. In the secondary analyses, we studied differences according to the educational level of the parents. To be able to directly compare the regression coefficients for different strategies, strategy scores were standardised. All regression analyses were controlled for the age and sex of the adolescent, the smoking status of the adolescent and the number of smoking parents (as reported by the adolescent). SEP differences in anti-smoking strategies are presented separately for strategies as reported by adolescents and strategies as reported by parents. The separate estimates were derived from an interaction term between SEP and a variable defining whether the respondent was an adolescent or parent. This interaction term tested for differences in these results. Finally, in a sensitivity analysis, we tested whether associations differed by the smoking status of adolescent by adding the interaction term SEP*adolescent smoking status.

## Results

Table 1 presents the mean of all anti-smoking parenting strategy scores as reported by adolescents and their parents. On the total anti-smoking strategy score, adolescent and parent scores were comparable. However, specific scores deviated, especially regarding the quality and frequency of communication. Parents reported higher communication scores than adolescents. In both adolescents and parents, scores were relatively high on warnings, home rules and reacting to the adolescent’s smoking behaviour.

Table 2 presents the characteristics of the adolescents and parents. More boys than girls participated. The male-to-female ratio decreased with educational track but was similar across parental educational levels. Fifty percent of the adolescents were 15 years old. The mean age was very similar across educational tracks and parental educational levels. Two thirds of the total population had never smoked, while 5 % were daily smokers. Smoking was substantially more prevalent in the low track (18.2 % smoking at least monthly) than in the middle (11.0 %) and high (10.6 %) tracks. In households with highly educated parents or an adolescent in a high educational track, parents were less often smokers.

**Table 2:** Characteristics of adolescents and parents by educational level. Percentages are calculated over the total population (*N* total)

ᅟ	Total	Adolescent educational track			Parental educational level		
		Low	Middle	High	Low	Middle	High
*N* total	452	231	246	198	39	105	234
*N* adolescents	225	77	82	66	13	35	78
*N* parents	227	154	164	132	26	70	156
Adolescent male sex (%)	57.8	61.0	57.3	54.5	53.8	62.9	52.6
Adolescent age (mean ± SD)	14.7 ± 0.73	14.5 ± 0.93	14.8 ± 0.56	14.7 ± 0.62	14.5 ± 0.78	14.7 ± 0.83	14.5 ± 0.64
13 or 14 (%)	39.1	53.2	25.6	39.4	46.2	40.0	50.0
15 (%)	50.2	29.9	65.9	54.4	46.2	48.6	44.9
16 or 17 (%)	10.7	16.9	8.5	6.1	7.7	11.4	5.1
Adolescent smoking status							
Never (%)	64.9	57.1	68.3	69.7	84.6	74.3	70.5
Tried a few times (%)	21.8	24.7	20.7	19.7	15.4	20.0	17.9
Monthly (%)	4.4	2.6	6.1	4.5	-	5.7	2.6
Weekly (%)	4.0	5.2	3.7	3.0	-	-	3.8
Daily (%)	4.9	10.4	1.2	3.0	-	-	5.1
Parental smoking reported by adolescent							
Neither (%)	65.3	53.2	68.3	75.8	53.8	74.3	74.4
One of them (%)	24.4	28.6	24.4	19.7	23.1	14.3	23.1
Both (%)	10.2	18.2	7.3	4.5	23.1	11.4	2.6

In Table 3, the mean scores of the strategies are presented according to the educational track of the adolescent and the educational level of the parents. There were no differences between educational tracks in the total anti-smoking strategy score. For specific anti-smoking strategies, the differences according to educational track and parental educational level were absent or small. Confidence intervals were overlapping in all cases.

**Table 3:** Mean scores with 95 % confidence interval of anti-smoking parenting strategies by educational level

Strategies	Adolescent educational track			Parental educational level		
	Low	Middle	High	Low	Middle	High
Communication quality	2.85 (2.67; 3.04)	2.84 (2.64; 3.04)	2.95 (2.78; 3.12)	2.89 (2.50; 3.27)	3.05 (2.83; 3.27)	3.02 (2.87; 3.17)
Monitoring child	3.56 (3.39; 3.74)	3.66 (3.47; 3.84)	3.53 (3.38; 3.69)	3.75 (3.44; 4.06)	3.58 (3.38; 3.78)	3.56 (3.43; 3.70)
Monitoring friends	3.42 (3.26; 3.58)	3.43 (3.24; 3.62)	3.24 (3.09; 3.39)	3.34 (3.02; 3.66)	3.24 (3.04; 3.45)	3.36 (3.22; 3.49)
Perceived influence	2.91 (2.71; 3.10)	3.02 (2.83; 3.21)	2.92 (2.76; 3.09)	2.89 (2.55; 2.23)	2.94 (2.73; 3.15)	2.86 (2.71; 3.00)
Negative reaction	3.28 (3.07; 3.48)	3.32 (3.13; 3.51)	3.29 (3.11; 3.46)	3.06 (2.61; 3.50)	3.25 (3.01; 3.48)	3.18 (3.03; 3.33)
Home rules	3.71 (3.51; 3.92)	3.95 (3.75; 3.14)	3.95 (3.77; 4.12)	3.71 (3.26; 4.15)	3.87 (3.66; 4.08)	3.96 (3.81; 4.11)
Warnings	4.14 (3.99; 4.29)	3.82 (3.64; 3.99)	4.09 (3.95; 4.23)	3.89 (3.51; 4.26)	4.18 (4.02; 4.34)	4.05 (3.92; 4.17)
No-smoking agreement	3.29 (3.08; 3.50)	3.33 (3.11; 3.54)	3.43 (3.23; 3.62)	3.11 (2.68; 3.55)	3.43 (3.17; 3.69)	3.27 (3.11; 3.43)
Reward	2.39 (2.19; 2.60)	2.21 (1.99; 2.43)	2.42 (2.21; 2.64)	2.22 (1.81; 2.63)	2.20 (1.95; 2.46)	2.31 (2.13; 2.48)
Communication frequency	2.84 (2.65; 3.02)	2.70 (2.52; 2.88)	2.80 (263; 2.96)	2.86 (2.51; 3.21)	2.74 (2.53; 2.95)	2.91 (2.78; 3.05)
Reacting to smoking	3.81 (3.63; 3.99)	3.97 (3.80; 4.15)	4.16 (4.03; 4.29)	3.83 (3.45; 4.21)	3.93 (3.72; 4.14)	4.12 (4.00; 4.23)
Total anti-smoking strategy score	3.24 (3.15; 3.34)	3.24 (3.14; 3.34)	3.26 (3.18; 3.34)	3.16 (2.97; 3.35)	3.24 (3.14; 3.35)	3.24 (3.18; 3.31)

All items were scored on a scale of 1–5, with 1 representing ‘strongly disagree’ and 5 representing ‘strongly agree’

^a^Mean scores with 95 % confidence intervals

Table 4 presents the associations between SEP and the anti-smoking parenting strategy scores. The regression coefficients (*β*) represent the increase or decrease in standardised parenting scores with a one-step increase in SEP. Results are presented according to the type of SEP measure (educational track of adolescent or educational level of parents). There were no consistent differences in the use of strategies according to the educational level of the parents or according to the educational track of the adolescent.

**Table 4:** Associations between SEP and anti-smoking parenting strategies for adolescent and parent reports of parenting strategies

Strategies	Adolescent educational track				*P* interaction^b^	Parental educational level				*P* interaction^b^
	Adolescents’ reports		Parents’ report			Adolescents’ reports		Parents’ report		
	*β* (95 % CI)^a^	*P* ^b^	*β* (95 % CI)^a^	*P* ^b^		*β* (95 % CI)^a^	*P* ^b^	*β* (95 % CI)^a^	*P* ^b^	
Communication quality	−0.04 (−0.23;0.33)	0.598	−0.07 (−0.24;0.10)	0.399	0.780	0.15 (−0.11;0.41)	0.255	0.02 (−0.20;0.23)	0.887	0.335
Monitoring child	−0.10 (−0.26;0.06)	0.234	0.05 (−0.12;0.22)	0.569	0.164	−0.03 (−0.29;0.24)	0.834	−0.05 (−0.27;0.16)	0.624	0.859
Monitoring friends	−0.10 (−0.27;0.06)	0.222	−0.06 (−0.22;0.11)	0.493	0.679	0.03 (−0.23;0.30)	0.800	−0.07 (−0.28;0.14)	0.502	0.496
Perceived influence	0.00 (−0.16;0.16)	0.991	0.01 (−0.15;0.18)	0.883	0.918	−0.06 (−0.32;0.19)	0.634	0.01 (−0.19;0.22)	0.900	0.613
Negative reaction	0.06 (−0.10;0.23)	0.466	−0.02 (−0.19;0.16)	0.849	0.432	−0.03 (−0.29;0.23)	0.824	−0.11 (−0.33;0.11)	0.343	0.561
Home rules	−0.03 (−0.20;0.13)	0.685	0.20 (0.03;0.37)	*0.022*	*0.023*	0.02 (−0.24;0.29)	0.858	0.08 (−0.15;0.31)	0.505	0.705
Warnings	−0.08 (−0.24;0.09)	0.357	−0.08 (−0.25;0.09)	0.341	0.966	0.17 (−0.09;0.43)	0.196	−0.17 (−0.39;0.04)	0.118	*0.013*
No-smoking agreement	−0.05 (−0.21;0.12)	0.566	0.17 (0.00;0.34)	*0.048*	*0.023*	−0.02 (−0.28;0.25)	0.903	−0.03 (−0.26;0.20)	0.797	0.920
Reward	−0.04 (−0.20;0.13)	0.678	0.01 (−0.16;0.19)	0.877	0.548	0.08 (−0.19;0.34)	0.573	−0.01 (−0.25;0.24)	0.960	0.436
Communication frequency	0.08 (−0.08;0.25)	0.326	−0.25 (−0.41;−0.08)	*0.004*	*0.002*	0.13 (−0.13;0.39)	0.320	0.03 (−0.18;0.24)	0.751	0.499
Reacting to smoking	0.11 (−0.06;0.27)	0.196	0.15 (−0.01;0.31)	0.067	0.486	0.04 (−0.22;0.29)	0.776	0.11 (−0.09;0.31)	0.266	0.618
Total anti-smoking strategy score	−0.03 (−0.19;0.14)	0.745	0.02 (−0.15;0.20)	0.813	0.604	0.08 (−0.18;0.35)	0.528	−0.06 (−0.29;0.17)	0.615	0.243

^a^Regression coefficient (*β*) with 95 % confidence interval for standardised parenting strategies. The regression coefficient represents the increase or decrease in parenting scores with a one-step increase in educational level

^b^ Italicalised *P*-values are significant at *α*=0.05

In Table 4, we tested whether associations between SEP and anti-smoking strategies were different when the strategies were reported by adolescents or parents. Overall, we found no consistent differences between adolescents’ and parents’ reports. For three anti-smoking strategies, the association with adolescent educational track was significantly stronger when reported by parents than it was when reported by adolescents: home rules, no-smoking agreement and communication frequency. These results suggest that parents of adolescents in higher educational tracks more often report to set home rules and to have a no-smoking agreement, but that they less often report to communicate about smoking with their children. These differences were not found according to the parental educational level (Table 4).

In sensitivity analyses, we tested whether associations between SEP and anti-smoking strategies were different for adolescents who were smokers and non-smokers. Overall, we did not find consistent differences between smokers and non-smokers. However, if their child did smoke, highly educated parents were less likely than lower educated parents to report a negative reaction to their child’s smoking (*β* = −0.55, 95 % confidence interval (CI) = −0.98; −0.12, *p* = 0.012, *p* for interaction = 0.026), and they perceived their influence to be weaker (*β* = −0.40, 95 % CI = −0.77; −0.02, *p* = 0.040, *p* for interaction = 0.026) (these results are not presented in the tables).

## Discussion

### Key Results

We found that there were no consistent socioeconomic differences in the use of anti-smoking parenting strategies. There were no statistically significant differences in relation to parental educational level or when using adolescent reports on parenting practices. However, when using parental reports, a few strategies varied significantly according to adolescent educational track. Adolescents in a higher educational track were more likely to have no-smoking rules in their home. They were also more likely to have a no-smoking agreement but significantly less likely to frequently communicate about smoking with their parents.

### Evaluation of Potential Limitations

The response among the parents was much higher in the high educational level group (90 %) than in the low educational level group (43 %). Parental response was also higher in parents who did not smoke (according to adolescent reports) and in non-smoking adolescents. The selective response might have biased our results if the response was associated with characteristics that were not controlled for in our study and that were related to parents’ anti-smoking practices.

This study used the educational level of adolescents and parents as indicators of SEP. The use of educational level has the advantages that it can easily be measured in both adolescents and adults and that respondents do not regard it as sensitive information. Furthermore, parental educational level as well as adolescent educational track has been shown to be highly predictive of smoking behaviour in young people [4, 7, 22]. We did not measure parents’ occupational status or income or the family affluence scale [23], and we cannot exclude the possibility that using these other SEP indicators would have revealed inequalities that were not found in this study.

It is uncertain whether the results can be generalised to the entire Dutch adolescent population. The secondary school selected for this study was situated in a middle-sized city in a central region of the country, with an income level close to the country average. The school included the three most important educational tracks and was of a medium size in terms of the number of students. The percentage of students with an immigrant background was however lower than average: around 5 % as compared to 20 % in the general Dutch population. Studies with larger and more diverse samples are needed to assess the generalizability our findings for the Netherlands and for other European countries.

### Interpretation of Results

We found discrepancies between parents and adolescents in their reports of the use of anti-smoking parenting strategies, especially for communication. While some previous studies have found adolescent and parent reports to be very similar [11], other studies have found discrepancies [24, 25]. Parents may over-report their use of strategies in order to come across as good parents. Adolescents, on the other hand, may not be aware of all strategies applied by the parents. Views may differ especially on the quality of communication, as adolescents may not be overly interested in their parents’ opinion on smoking, while the parents may be highly interested in their child’s experiences. As parents’ and adolescents’ reports reflect different viewpoints, we included both reports in this study.

We found that parents more frequently talked about smoking with their children when their children were from lower educational tracks but not when they themselves had a lower educational level. Parents of children in higher educational tracks may put more trust in the judgement of their child and do not as much try to influence their decision-making [26]. In addition, parents of adolescents in lower educational tracks may be inclined to communicate more frequently about smoking, because of the higher smoking prevalence in low tracks compared to high tracks [4, 5]. A Dutch longitudinal study observed that the frequency of communication increased if adolescents started smoking [25].

Socioeconomic inequalities in adolescent smoking may only be influenced by inequalities in anti-smoking parenting strategies if these strategies have an effect on adolescent smoking behaviour. The evidence on such effects is however mixed. For the following strategies, the literature provides evidence of an effect on adolescent smoking behaviour: parental monitoring of (smoking) behaviour [20, 21, 27], smoking restrictions in the home [28], high quality of communication [11, 29], no-smoking agreements [25] and the perception of high parental influence [11]. We found differences by SEP for only two of these strategies: smoking restrictions in the home and no-smoking agreements. These two strategies will be discussed below.

Our results suggest that parents of adolescents in lower educational tracks tended to be less likely to set no-smoking rules in their home. This was only found according to the parent reports and not according to the adolescent reports. This finding bares resemblance to a study by Bolte et al. in which lower educated parents were less likely to have a smoke-free home in households with smokers [30]. As a consequence, children with a lower educational level were more often exposed to smoking in the home. A 2010 review demonstrated that smoking restrictions in the home were associated with reduced adolescent smoking behaviours, especially when the home is completely smoke-free [28]. Less exposure to smoking rules in the homes of lower SEP adolescents rules may contribute to the higher smoking prevalence in lower educated adolescents.

Parents of adolescents in higher educational tracks were more likely to have a no-smoking agreement with their child. Harakeh et al. [11] and Huver et al. [31] did not find an association between having a no-smoking agreement and adolescent smoking behaviour or smoking cognitions. However, a longitudinal study by De Leeuw et al. [25] found that, compared to non-smokers, stable smokers were less likely to have a no-smoking agreement with their parents and that those who decreased their smoking behaviour over time were more likely to have an established agreement [25]. If no-smoking agreements do affect adolescent smoking trajectories, adolescents in higher educational tracks may benefit from more often having no-smoking agreements with their parents.

Despite the seemingly modest role of anti-smoking parenting strategies, SEP inequalities in adolescent smoking may be influenced by parents through other mechanisms. Parental smoking is an important risk factor for adolescent smoking initiation [8], and since parents of low SES are more often smokers, lower SEP adolescent are more likely to be exposed to parental smoking. Some studies found that the parenting style is more important for the prevention of smoking than anti-smoking strategies [13, 32–35]. Parenting styles characterised by warmth, care and positive emotional attachment are associated with lower smoking rates [13, 32, 33, 35]. Authoritarian, permissive and neglectful parenting styles are associated with higher odds of smoking [32]. Authoritarian or autocratic parenting styles have previously been found to be more common in parents with lower income [26], with lower educational levels [36, 37] and with lower occupational status [38].

### Conclusions

We did not identify consistent socioeconomic differences in the use of parenting strategies to prevent adolescent smoking. In this specific population, we therefore found very limited support for the hypothesis that anti-smoking parenting strategies contribute to socioeconomic inequalities in adolescent smoking. This suggests that if parents’ behaviours contribute to inequalities in adolescent smoking, the use of different anti-smoking parenting strategies is less important than the parents’ own smoking behaviour and their general parenting style.
